# SUDEP kompakt – praxisrelevante Erkenntnisse und Empfehlungen zum plötzlichen, unerwarteten Tod bei Epilepsie

**DOI:** 10.1007/s00115-021-01075-3

**Published:** 2021-02-16

**Authors:** Rainer Surges, Stefan Conrad, Hajo M. Hamer, Andreas Schulze-Bonhage, Anke M. Staack, Bernhard J. Steinhoff, Adam Strzelczyk, Eugen Trinka

**Affiliations:** 1 Klinik und Poliklinik für Epileptologie, Universitätsklinikum Bonn, Venusberg-Campus 1, 53127 Bonn, Deutschland; 2 Deutsche Epilepsievereinigung, Berlin, Deutschland; 3 Epilepsiezentrum, Klinik für Neurologie, Universitätsklinikum Erlangen, Erlangen, Deutschland; 4 Epilepsiezentrum, Universitätsklinikum Freiburg, Freiburg, Deutschland; 5 Epilepsiezentrum Kork, Kehl-Kork, Deutschland; 6 Universitätsklinik Freiburg, Freiburg, Deutschland; 7 Epilepsiezentrum Frankfurt Rhein-Main, Zentrum der Neurologie und Neurochirurgie, Goethe-Universität Frankfurt, Frankfurt am Main, Deutschland; 8 Department of Neurology, Christian Doppler Klinik, Paracelsus Medical University and Centre for Cognitive Neuroscience, Salzburg, Österreich; 9 Department of Public Health, Health Services Research and Health Technology Assessment, UMIT – University for Health Sciences, Medical Informatics and Technology, Hall in Tirol, Österreich

**Keywords:** Plötzlicher Tod bei Epilepsie, Asystolie, Wearables, Vorzeitige Sterblichkeit, Aufklärung, Sudden death in epilepsy, Asystole, Wearables, Premature mortality, Counselling

## Abstract

„Sudden unexpected death in epilepsy“ (SUDEP) ist der plötzliche, unerwartete Tod eines Epilepsiepatienten, der unter „gutartigen“ Umständen und ohne typische Todesursachen auftritt. SUDEP betrifft alle Epilepsiepatienten. Das individuelle Risiko hängt vor allem von Merkmalen der Epilepsie und Anfälle sowie von Lebensumständen ab. Fokale zu bilateral bzw. generalisierte tonisch-klonische Anfälle (TKA), nächtliche Anfälle und fehlende nächtliche Überwachung erhöhen das Risiko. In den meisten SUDEP-Fällen kommt es nach TKA zu einer fatalen Kaskade mit Apnoe, Hypoxämie und Asystolie. Wahrscheinlich könnten zwei Drittel der SUDEP-Fälle bei nicht überwachten Epilepsiepatienten mit TKA verhindert werden. Mobile Geräte („wearables“) können nächtliche TKA erkennen und Hilfspersonen benachrichtigen. Eine SUDEP-Aufklärung wird von den meisten Patienten und Angehörigen gewünscht, beeinflusst Therapieadhärenz und Verhalten günstig und hat keine negativen Auswirkungen auf Stimmung oder Lebensqualität.

Empfehlungen der Kommission „Patientensicherheit“ der Deutschen Gesellschaft für Epileptologie: Therapieziel ist Anfallsfreiheit. Wenn dies nicht möglich ist, soll versucht werden, zumindest TKA zu kontrollieren. Alle Epilepsiepatienten und ihre Angehörigen sollen über SUDEP und Risikofaktoren aufgeklärt werden. Patienten und Angehörige sollen über Maßnahmen informiert werden, die einem erhöhten Risiko bzw. einem drohenden SUDEP entgegenwirken. Die Aufklärung soll in einem persönlichen Gespräch erfolgen, bei Diagnosestellung oder später. Die Aufklärung sollte dokumentiert werden. *Wearables* zur Detektion von TKA können empfohlen werden. Bei persistierenden TKA sollen Therapieversuche zur Anfallskontrolle fortgeführt werden. Nach SUDEP sollten Hinterbliebene kontaktiert werden.

## Abstract (en)

Sudden unexpected death in epilepsy (SUDEP) is the sudden and unexpected death of an epilepsy patient, which occurs under benign circumstances without evidence of typical causes of death. SUDEP concerns all epilepsy patients. The individual risk depends on the characteristics of the epilepsy and seizures as well as on living conditions. Focal to bilateral and generalized tonic-clonic seizures (TCS), nocturnal seizures and lack of nocturnal supervision increase the risk. Most SUDEP cases are due to a fatal cascade of apnea, hypoxemia and asystole in the aftermath of a TCS. Two thirds of SUDEP cases in unsupervised epilepsy patients with TCS could probably be prevented. Wearables can detect TCS and alert caregivers. SUDEP information is desired by most patients and relatives, has a favorable impact on treatment adherence and behavior and has no negative effects on mood and quality of life.

Recommendations of the committee on patient safety of the German Society of Epileptology: the ultimate treatment goal is seizure freedom. If this cannot be achieved, control of TCS should be sought. All epilepsy patients and their relatives should be informed about SUDEP and risk factors. Patients and relatives should be informed about measures to counteract the elevated risk and imminent SUDEP. The counselling should be performed during a face-to-face discussion, at the time of first diagnosis or during follow-up visits. The counselling should be documented. Wearables for TCS detection can be recommended. If TCS persist, therapeutic efforts should be continued. The bereaved should be contacted after a SUDEP.

## Hintergrund

Menschen mit Epilepsie haben im Vergleich zur Allgemeinbevölkerung ein 24-fach erhöhtes Risiko, plötzlich und unerwartet zu versterben [4]. Bei einer seit Kindes- und Jugendalter bestehenden Epilepsie beträgt das kumulative Lebenszeitrisiko eines plötzlichen Todes insgesamt 7–8 % [38]. Trotz dieser eindrücklichen Zahlen sind nur wenige Betroffene oder Angehörige informiert [33], und die Mehrheit der behandelnden Fachärzte spricht nie oder nur selten über den plötzlichen und unerwarteten Tod bei Epilepsie (abgekürzt SUDEP; [32]). Die ausbleibende Diskussion über SUDEP beruht unter anderem auf den Annahmen, dass eine Aufklärung keine praktischen Konsequenzen für Präventionsmaßnahmen hat und dass dadurch bei Betroffenen und Angehörigen zusätzlich Stress und Ängste ausgelöst werden [32]. Im Gegensatz dazu legen neue Erkenntnisse zu Pathophysiologie und Risikofaktoren sowie Fortschritte bei mobilen Gesundheitstechnologien nahe, dass die Aufklärung über SUDEP zu einer Reduktion des Risikos und der Inzidenz beitragen kann. Der vorliegende Artikel wurde von den Mitgliedern der Kommission „Patientensicherheit“ der Deutschen Gesellschaft für Epileptologie daher mit dem Ziel verfasst, eine Auswahl der wichtigsten Informationen und Aspekte zum SUDEP kompakt darzustellen und daraus Empfehlungen für den Alltag in Klinik und Praxis verständlich abzuleiten und zu erklären.

## Definition

SUDEP ist ein Akronym aus dem angloamerikanischen Sprachraum und steht für „sudden unexpected death in epilepsy“. Vereinfacht definiert ist SUDEP der plötzliche und unerwartete Tod eines Menschen mit Epilepsie, der unter „gutartigen“ Umständen aufgetreten ist und nicht durch andere innere oder äußere Faktoren verursacht wurde [17]. Eine differenzierte Klassifikation in definitiver, wahrscheinlicher und möglicher SUDEP bzw. SUDEP-Plus (bei bestehender Vorerkrankung, die prinzipiell zum plötzlichen Tod führen kann, jedoch dafür keine Hinweise vorliegen) ist erst nach einer Autopsie möglich.

### Hinweis:

In Deutschland werden bei Epilepsiepatienten oft keine Autopsien durchgeführt, sodass die Diagnosestellung eines definitiven SUDEP selten ist.

## Inzidenzrate

Die Angaben zur Häufigkeit des SUDEP sind aus verschiedenen Gründen heterogen und variieren bei Erwachsenen (ab 18 Jahren) um 1 pro 1000 Personenjahre, bei Patienten mit schwer behandelbarer Epilepsie bzw. epilepsiechirurgischen Kandidaten wurden auch Inzidenzraten um 7 pro 1000 Personenjahre publiziert [39]. Bei Kindern und Jugendlichen wurden ehemals geringere Inzidenzraten angenommen (siehe auch [6]), aber in neueren Arbeiten aus Kanada und Schweden wurde eine SUDEP-Inzidenz von 1,1 pro 1000 Personenjahre berechnet [10, 35]. Auch bei Kindern und Jugendlichen ist davon auszugehen, dass das individuelle Risiko von der Schwere der Epilepsie abhängt und beispielsweise Patienten mit einem schwer behandelbaren Dravet-Syndrom (genetisch bedingte epileptische Enzephalopathie) ein höheres SUDEP-Risiko haben als weniger schwere Epilepsieformen.

### Hinweis:

Auch bei vermeintlich „benignen“ Epilepsien kann es zu einem SUDEP kommen [44], und sogar zu Beginn einer Epilepsieerkrankung wurden mittlerweile SUDEP-Fälle berichtet [7].

## Pathophysiologie

**Figure 1:**
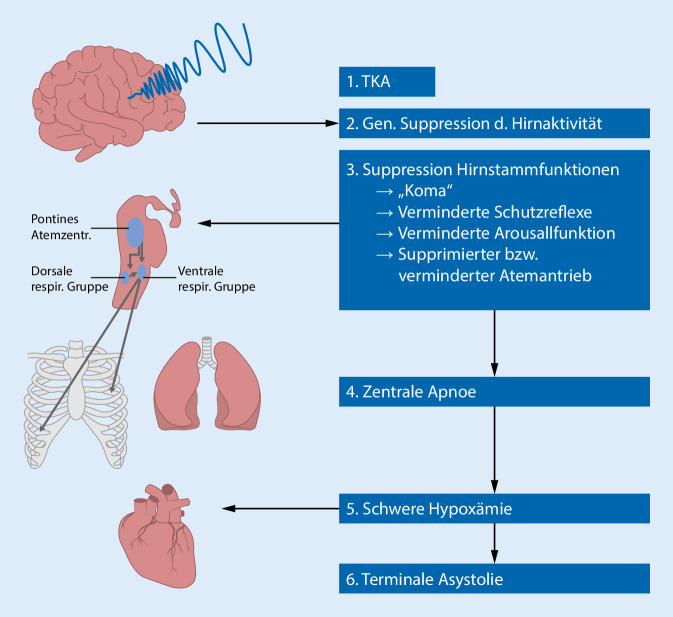

Die überwiegende Zahl der SUDEP-Fälle tritt wahrscheinlich unmittelbar im zeitlichen Zusammenhang mit epileptischen Anfällen auf [40]. In der MORTEMUS-Studie wurden weltweit SUDEP-Fälle zusammengetragen, die während Video-EEG-Langzeitableitungen aufgetreten sind [26]. Allen in der MORTEMUS-Studie dokumentierten SUDEP-Fällen ging ein generalisierter bzw. fokaler zu bilateral tonisch-klonischer Anfall (TKA) voraus. Aus dieser Studie und anderen Fallberichten lässt sich eine typische SUDEP-Kaskade (Abb. 1) ableiten, bei der es in der frühen postiktualen Phase eines TKA zu einer generalisierten Hemmung der Hirnaktivität kommt, die auf Hirnstammebene zu einem zentralen Atemstillstand führt. Tiermodelle legen nahe, dass die anfallsgetriggerte Suppression der Hirnaktivität durch eine kortikale Depolarisationswelle verursacht wird, die zum Hirnstamm propagiert und dort neuronale Aktivität hemmt. Zudem wird vermutet, dass endogene Opioide, Adenosin und Serotonin eine Rolle in der Vermittlung der postiktualen Atemstörung spielen [19]. Zur Verschlimmerung der postiktualen Atemstörung trägt auch die Bauchlage bei, die bei etwa 75 % der SUDEP-Fälle beobachtet wurde [14] und die durch Verlegung der Atemwege und Behinderung der Atemexkursionen die Sauerstoffversorgung und das Abatmen von Kohlendioxid zusätzlich kompromittieren. Die aus dem Atemstillstand resultierende Hypoxämie wiederum verursacht durch direkte Effekte auf das Herz eine Bradykardie bzw. eine terminale Asystolie [19, 26]. Sehr viel seltener treten anfallsassoziierte ventrikuläre Tachyarrhythmien als Ursache eines SUDEP auf [34]. Bei einem kleinen Anteil der SUDEP-Ereignisse lassen sich keine Anzeichen eines zeitlich assoziierten epileptischen Anfalls ausmachen [40]. In diesen Fällen werden vor allem interiktuale fatale Herzrhythmusstörungen vermutet, die u. a. durch Antiepileptikaeinnahme und die vermutlich durch die chronische Epilepsie bedingt veränderten Herzeigenschaften (Konzept des sog. „epileptischen Herzens“) begünstigt werden können [19, 34].

### Hinweis:

Der Atemstillstand nach Sistieren des TKA scheint das zentrale Element in der Pathophysiologie der meisten SUDEP-Fälle zu sein, die postiktuale Asystolie tritt erst sekundär auf. Iktuale (also während des Anfalls auftretende) Asystolien kommen meist bei Schläfenlappenepilepsien vor und stehen wahrscheinlich nicht in ursächlichem Zusammenhang mit SUDEP. Iktuale Asystolien haben ein hohes Rezidivrisiko. Sollte bei Betroffenen mit iktualen Asystolien keine Anfallsfreiheit erreicht werden, kann die Herzschrittmacherimplantation vor synkopenbedingten Stürzen und Verletzungen schützen [34].

## Risikofaktoren

In epidemiologischen Studien wurde eine Reihe von Risikofaktoren für SUDEP identifiziert [39, 40]. Die wichtigsten Risikofaktoren, gemessen an der vermuteten Effektstärke und Reproduzierbarkeit in verschiedenen Studien, sind das Auftreten und die Häufigkeit von TKA sowie das Auftreten nächtlicher Anfälle bzw. nächtlicher TKA [12, 36]. Passend zu diesen Befunden ist, dass auch eine unzureichende Therapieadhärenz mit einem erhöhten SUDEP-Risiko assoziiert ist [37]. Das Fehlen einer nächtlichen Überwachung [13, 36, 42] und die Bauchlage der Verstorbenen beim Auffinden wurden ebenfalls häufig bei SUDEP-Fällen dokumentiert [14].

### Hinweis:

Die wichtigsten Risikofaktoren sind prinzipiell modifizierbar (Tab. 1) und können demnach wahrscheinlich günstig beeinflusst werden. Die Absicherung einer (vor allem auch nächtlichen) Anfallskontrolle durch Video-EEG-Monitoring oder mobile EEG-Verfahren kann zur Therapiesteuerung und Minimierung des SUDEP-Risikos beitragen. Eine Bauchlage zu Beginn eines TKA erhöht das Risiko zum Ende des Anfalls in dieser zu verbleiben, sodass die Vermeidung von Bauchlage beim Schlafen empfohlen werden kann [16].

**Table 1:** 

Risikofaktor	Odds Ratio^a^ (95 %-KI)	Referenz
*TKA in letzten 3 Monaten*	13,8 (6,6–29,1)	[13]
*Häufigkeit von TKA pro Jahr*		
1–2	2,9 (n.a.)	[8]
3–12	8,3 (n.a.)	[8]
13–50	9,1 (n.a.)	[8]
> 50	14,5 (n.a.)	[8]
*(Vorangegangene) nächtliche Anfälle*	3,9 (2,5–6,0)	[12]
*Fehlende nächtliche Überwachung (in Einrichtung allein schlafen)*	2,5 (1,25–5)	[13]
*Fehlende nächtliche Überwachung (allein leben)*	5,0 (2,93–8,57)	[36]
*Fehlende nächtliche Überwachung und TKA*	67,1 (29,7–151,9)	[36]

*n.a.* nicht angegeben

^a^ Die Odds Ratio ist hier ein Maß dafür, um wie viel größer die Chance in der Gruppe mit dem Risikofaktor ist, an SUDEP zu versterben, verglichen mit der Chance in der Gruppe ohne Risikofaktor

## Prävention und Risikoreduktion

Eine wirksame antiepileptische Pharmakotherapie reduziert signifikant das SUDEP-Risiko [25, 37]. Weder Polypharmakotherapie noch einzelne Substanzen scheinen *per se* das SUDEP-Risiko zu erhöhen [9, 19, 37]. Auch bei nichtmedikamentösen Therapieverfahren wie der resektiven Epilepsiechirurgie, der Vagusnervstimulation und der tiefen Hirnstimulation der anterioren Thalamuskerne wurden vergleichsweise geringe SUDEP-Inzidenzraten beobachtet, sodass ein protektiver Effekt plausibel ist [27, 29, 31]. Geringere SUDEP-Inzidenzraten wurden ebenfalls beobachtet, wenn nächtliche Überwachungsmaßnahmen wie akustische Geräte (Babyphone), Bewegungssensoren am Bett und eine Videoüberwachung zur Anwendung kamen oder Zimmernachbarn anwesend waren [13, 42]. Schätzungen zufolge könnten etwa zwei Drittel der SUDEP-Fälle bei alleinlebenden Epilepsiepatienten mit TKA verhindert werden, wenn die TKA vollständig kontrolliert wären oder eine nächtliche Überwachung vorhanden wäre [36].

### Hinweis:

Bei Durchführung einfacher Maßnahmen wie Berühren oder Lagerung (in die stabile Seitenlage) von Epilepsiepatienten nach stattgehabtem TKA wurde eine kürzere Dauer der anfallsassoziierten Atmungsstörung und der postiktualen Bewegungslosigkeit gefunden [24]. Bei eingetretenem Atem- und Kreislaufstillstand nach TKA scheint eine frühzeitig einsetzende kardiopulmonale Reanimation einen SUDEP verhindern zu können [26].

## Technische Hilfsmittel zur nächtlichen Überwachung

Das unbeobachtete Auftreten von (nächtlichen) TKA mit nachfolgender fataler SUDEP-Kaskade ist wahrscheinlich der wichtigste Faktor in den meisten SUDEP-Fällen. Eine notfallmäßige Benachrichtigung formell oder informell Pflegender (Angehörige, Partner, Freunde) könnte die frühzeitige Durchführung einfacher Interventionen, die Überprüfung der Atemtätigkeit und ggf. frühe kardiopulmonale Wiederbelebungsmaßnahmen erlauben. Daher ist die Annahme plausibel, dass der Einsatz technischer Hilfsmittel zur Echtzeiterkennung von TKA das SUDEP-Risiko reduzieren kann.

Zur zuverlässigen und alltagstauglichen Detektion nächtlicher TKA wurden bereits einige tragbare Sensoren („wearables“) erfolgreich prospektiver klinischer Prüfungen unterzogen [2, 28, 43]. Dabei wurden Geräte eingesetzt, die die typischen TKA-assoziierten rhythmischen Kloni per Akzelerometrie, Druck (Matratzensensor) oder Elektromyographie bzw. die tonische Muskelversteifung per Elektromyographie erfassen können.

### Hinweis:

Die *International League against Epilepsy* und *International Federation of Clinical Neurophysiology* erklären, dass Geräte zur Detektion von TKA empfohlen werden können, vor allem dann, wenn daraus eine zeitnahe Intervention resultiert [2]. Einige dieser Geräte sind mittlerweile auch in Deutschland kommerziell erhältlich. Die Kostenübernahme durch die Krankenkassen ist bislang noch nicht einheitlich geregelt, sondern erfordert einen individuellen Antrag zur Kostenübernahme. Es muss im Einzelfall mit den Betroffenen zwischen der Kontrolle durch eine nächtliche Überwachung und deren Einschränkung der Autonomie des Betroffenen abgewogen werden.

## Aufklärung über SUDEP

Umfragen aus dem deutschsprachigen Raum haben gezeigt, dass auch an Epilepsiezentren behandelte Patienten nur selten von SUDEP gehört haben und dass, passend zu dieser Beobachtung, etwa zwei Drittel der befragten Neuropädiater bzw. Neurologen mit ihren Patienten selten oder nie über SUDEP sprechen [32, 33]. Im Kontrast dazu steht, dass die meisten Patienten und Angehörige zumindest grundlegend über das SUDEP-Risiko informiert werden möchten [5, 22, 23, 45] und sich etwa drei Viertel der Hinterbliebenen von an SUDEP verstorbenen Epilepsiepatienten vor dem Tod ein Gespräch über SUDEP gewünscht hätten [15]. Die Mehrzahl der Patienten oder Angehörige und Partner wünscht sich eine SUDEP-Aufklärung idealerweise bei Stellung der Epilepsiediagnose oder kurze Zeit später, in einem persönlichen Gespräch mit dem Arzt oder Epilepsiefachassistenten [5, 11, 21–23, 41, 45]. Die Information über SUDEP und die Risikofaktoren führt zu Verhaltensänderungen, die günstige Effekte auf einzelne modifizierbare Risikofaktoren haben (z. B. Therapieadhärenz, Anfallshäufigkeit [20, 30]).

### Hinweis:

Erst das Wissen um SUDEP ermöglicht es Betroffenen und Angehörigen, Maßnahmen oder Verhaltensänderungen zu ergreifen, die das Risiko vermindern können. Eine erhöhte Ängstlichkeit, Niedergestimmtheit oder verminderte Lebensqualität durch die Aufklärung über SUDEP ist laut Studienlage weder bei Angehörigen noch Betroffenen zu erwarten [5, 20, 41]. Eine Kontaktaufnahme nach einem SUDEP wird von vielen Hinterbliebenen gewünscht und kann bei der Trauerverarbeitung unterstützen [1, 3, 18].

## Empfehlungen für die Praxis

- Alle Epilepsiepatienten sowie Angehörige und Partner sollen über SUDEP und Risikofaktoren aufgeklärt werden.
- Die Aufklärung soll in einem persönlichen Gespräch zu einem frühen Zeitpunkt stattfinden, d. h. am besten bei der Diagnosestellung oder dem ersten Kontrolltermin.
- Patienten, Angehörige und Lebenspartner sollen über Maßnahmen informiert werden, die einem erhöhten Risiko bzw. einem drohenden SUDEP entgegenwirken können (siehe Infobox 1).
  - Zu den modifizierbaren Risikofaktoren bzw. begünstigenden Faktoren, auf die Betroffene selbst einwirken können, zählen v. a. eine verbesserte Anfallskontrolle, Therapieadhärenz, nächtliche Überwachungsmaßnahmen z. B. durch mobile Gesundheitstechnologien, vorzugsweise in Gemeinschaft leben und Vermeidung bekannter Anfallsauslöser (z. B. Schlafentzug, Alkoholkonsum).
  - Angehörigen, Partnern und Pflegenden können einfache Hilfsmaßnahmen wie stabile Seitenlagerung nach TKA, Überprüfung der Atmung und des Pulses, Überwachung für etwa 60 min nach Anfallsende sowie ein regelmäßiges Training der kardiopulmonalen Reanimationsmaßnahmen empfohlen werden.
- Das Gespräch sollte schriftlich dokumentiert werden.
- Klinisch adäquat geprüfte Geräte zur Detektion von TKA können empfohlen werden, vor allem dann, wenn daraus eine zeitnahe Intervention resultieren kann.
- Bei Patienten mit persistierenden TKA sollen Therapieversuche zur Anfallskontrolle aktiv fortgeführt werden.
- Pharmakoresistente Patienten sollten frühzeitig in ein epilepsiechirurgisch tätiges Behandlungszentrum überwiesen werden, um die Indikation für nichtmedikamentöse Therapieverfahren zu prüfen.
- Nach vermutetem SUDEP sollte Kontakt mit den Hinterbliebenen aufgenommen werden.

### Infobox 1 Mögliche Formulierungen für das Gespräch über SUDEP

„Sie fragen sich vielleicht, ob Epilepsien gefährlich sein können. Es gibt bekanntlich das Risiko von Verletzungen oder Unfällen bei einem epileptischen Anfall.“

„Man kann auch direkt an epileptischen Anfällen versterben. Dieses Risiko ist aber gering.“

„Bei tonisch-klonischen Anfällen, also wenn sich der ganze Körper zunächst versteift und anschließend rhythmisch zuckt, ist dieses Risiko am höchsten. In sehr seltenen Fällen kann es nämlich passieren, dass es nach einem solchen Anfall zu einem Atemstillstand kommt. Das ist die Hauptursache für den sog. SUDEP, also dem plötzlichen, unerwarteten Tod bei Epilepsie.“

„Das SUDEP-Risiko für einen einzelnen Anfall kann nicht genau benannt werden, es ist aber sehr gering.“

„SUDEP tritt typischerweise bei einem von 1000 Menschen mit Epilepsie in einem Jahr auf. Das heißt auch, dass jährlich 999 von 1000 Menschen mit Epilepsie nicht an einem SUDEP sterben.“

„Bei manchen Menschen mit Epilepsie ist das Risiko höher als bei anderen. Das hängt vor allem von der Schwere und Häufigkeit der Anfälle und den Lebensumständen ab.“

„Die wichtigsten Risikofaktoren sind das Auftreten nächtlicher Anfälle und die Häufigkeit tonisch-klonischer Anfälle.“

„Anfallsfreiheit und vor allem die vollständige Kontrolle tonisch-klonischer Anfälle ist stark mit einem verminderten SUDEP-Risiko verbunden.“

„Wie kann man das Risiko für einen SUDEP vermindern? Alle Maßnahmen, die zu einer besseren Anfallskontrolle führen, sind hilfreich. Dazu zählen eine regelmäßige Einnahme der Tabletten, ein guter Schlaf und das Vermeiden von Schlafentzug sowie kein oder nur geringer Alkoholkonsum. Bei Zunahme der Häufigkeit oder Schwere der Anfälle melden Sie sich bei mir.“

„Ein weiterer wichtiger Risikofaktor ist die fehlende Beobachtung vor allem nächtlicher Anfälle. Das unbemerkte Auftreten von Anfällen, vor allem nachts, ist ein großes Problem, da in diesen Fällen keine Hilfe durch andere geleistet werden kann.“

„Es sind aber mittlerweile kleine Geräte zur nächtlichen Überwachung erhältlich, mit denen tonisch-klonische Anfälle zuverlässig erkannt werden können und die im Notfall Angehörige und Partner benachrichtigen können. Diese Geräte stören den Nachtschlaf nicht und schränken die Intimsphäre nicht ein. Wägen Sie die möglichen Vorteile einer nächtlichen Überwachung gegen die möglichen Nachteile ab.“

„Als Familie/Lebenspartner sollten Sie wissen, dass tonisch-klonische Anfälle meist nach 2–3 min wieder von selbst aufhören. Wichtig ist dabei, Verletzungen durch Stürze oder nahe stehende Gegenstände zu vermeiden und darauf zu achten, dass nach Anfallsende die Atmung wieder einsetzt. Das hört man auch an den schnaufend-röchelnden Atemgeräuschen, die typischerweise nach tonisch-klonischen Anfällen einsetzen. Achten Sie für mindestens 45–60 min nach dem Anfall auf die Atmung und den Puls.“

„Nehmen Sie an einem Erste-Hilfe-Kurs teil und wiederholen sie dieses Training regelmäßig, z. B. alle 2 Jahre.“

„Ich fasse kurz zusammen: Es besteht ein geringes Risiko, plötzlich an Epilepsie zu versterben. Eine gute Anfallskontrolle, eine regelmäßige Medikamenteneinnahme und das Vermeiden typischer Anfallsauslöser können das Risiko stark vermindern. Haben Sie weitere Fragen?“
